# Declines in ice cover are accompanied by light limitation responses and community change in freshwater diatoms

**DOI:** 10.1093/ismejo/wrad015

**Published:** 2024-01-10

**Authors:** Brittany N Zepernick, Emily E Chase, Elizabeth R Denison, Naomi E Gilbert, Alexander R Truchon, Thijs Frenken, William R Cody, Robbie M Martin, Justin D Chaffin, George S Bullerjahn, R Michael L McKay, Steven W Wilhelm

**Affiliations:** Department of Microbiology, The University of Tennessee, Knoxville, TN 37996, United States; Department of Microbiology, The University of Tennessee, Knoxville, TN 37996, United States; Department of Microbiology, The University of Tennessee, Knoxville, TN 37996, United States; Department of Microbiology, The University of Tennessee, Knoxville, TN 37996, United States; Lawrence Livermore National Laboratory, Livermore, CA 94550, United States; Department of Microbiology, The University of Tennessee, Knoxville, TN 37996, United States; HAS University of Applied Sciences, 5223 DE ‘s-Hertogenbosch, The Netherlands; Great Lakes Institute for Environmental Research, University of Windsor, Windsor, Ontario, N9C 1A2, Canada; Aquatic Taxonomy Specialists, Malinta, OH 43535, United States; Department of Microbiology, The University of Tennessee, Knoxville, TN 37996, United States; Stone Laboratory and Ohio Sea Grant, The Ohio State University, Put-In-Bay, OH 43456, United States; Department of Biological Sciences, Bowling Green State University, Bowling Green, OH 43403, United States; Great Lakes Institute for Environmental Research, University of Windsor, Windsor, Ontario, N9C 1A2, Canada; Department of Microbiology, The University of Tennessee, Knoxville, TN 37996, United States

**Keywords:** winter limnology, Great Lakes, climate change, proton-pumping rhodopsins, fasciclins

## Abstract

The rediscovery of diatom blooms embedded within and beneath the Lake Erie ice cover (2007–2012) ignited interest in psychrophilic adaptations and winter limnology. Subsequent studies determined the vital role ice plays in winter diatom ecophysiology as diatoms partition to the underside of ice, thereby fixing their location within the photic zone. Yet, climate change has led to widespread ice decline across the Great Lakes, with Lake Erie presenting a nearly “ice-free” state in several recent winters. It has been hypothesized that the resultant turbid, isothermal water column induces light limitation amongst winter diatoms and thus serves as a competitive disadvantage. To investigate this hypothesis, we conducted a physiochemical and metatranscriptomic survey that spanned spatial, temporal, and climatic gradients of the winter Lake Erie water column (2019–2020). Our results suggest that ice-free conditions decreased planktonic diatom bloom magnitude and altered diatom community composition. Diatoms increased their expression of various photosynthetic genes and iron transporters, which suggests that the diatoms are attempting to increase their quantity of photosystems and light-harvesting components (a well-defined indicator of light limitation). We identified two gene families which serve to increase diatom fitness in the turbid ice-free water column: proton-pumping rhodopsins (a potential second means of light-driven energy acquisition) and fasciclins (a means to “raft” together to increase buoyancy and co-locate to the surface to optimize light acquisition). With large-scale climatic changes already underway, our observations provide insight into how diatoms respond to the dynamic ice conditions of today and shed light on how they will fare in a climatically altered tomorrow.

## Introduction

Winter was historically considered a period of planktonic persistence rather than growth [1, 2]. Limnological surveys conducted in the winters of 2007–2012 contested this supposition with the rediscovery of dense diatom blooms associated with the ice cover of Lake Erie (US, Canada) [3, 4]. This finding ignited interest in winter limnology [5–7], with subsequent studies demonstrating that ice-associated communities were dominated by the centric colonial diatoms *Aulacoseira islandica* (of the class *Coscinodiscophyceae*) and *Stephanodiscus* spp. (of the class *Mediophyceae*) [3, 4, 8–10]. Chlorophyll *a* (Chl *a*) concentrations during winter surpassed those occurring in spring [4], and examinations of silica deposition in frustules demonstrated that cells were metabolically active [11]. Results of additional studies highlighted the biotic and biogeochemical importance of these blooms, as winter–spring diatom biovolumes surpass summer cyanobacterial biovolumes by 1.5- to 6-fold [12] and drive recurrent summer hypoxia in the Lake Erie central basin [6, 10, 12].

Though Lake Erie serves as a leading case study for winter diatom blooms, they are not an isolated phenomenon. Blooms often go unreported due to a lack of winter surveys [13], yet blooms have been well documented beneath the ice in Lake Baikal [14, 15] and characterized in other north temperate freshwater systems such as The Loch (US) [16], St. Lawrence River (Canada) [17], Lake Barleber (Germany) [13], Lakes Ladoga and Onega (Russia) [18], Lake Kasumigaura (Japan) [19], and the River Danube (Hungary) [20].

Contributing to the ecological success of winter diatoms are adaptations that increase membrane fluidity and enhance light harvesting in icy, low-light conditions [9]. Yet, arguably a major adaptation responsible for winter diatom success is their ability to partition to surface ice cover *via* interactions with ice-nucleating bacteria, a process that allows diatoms to maintain themselves under the ice surface and access optimal light for photosynthesis [8, 21]. Cumulatively, studies demonstrate ice cover plays a role in shaping winter diatom ecophysiology and increasing competitive fitness [3, 4, 8, 9, 11, 21]. However, this finding raises the question of how this keystone phylum [22–25] might fare in a climatically altered ice-free future.

**Figure 1:**
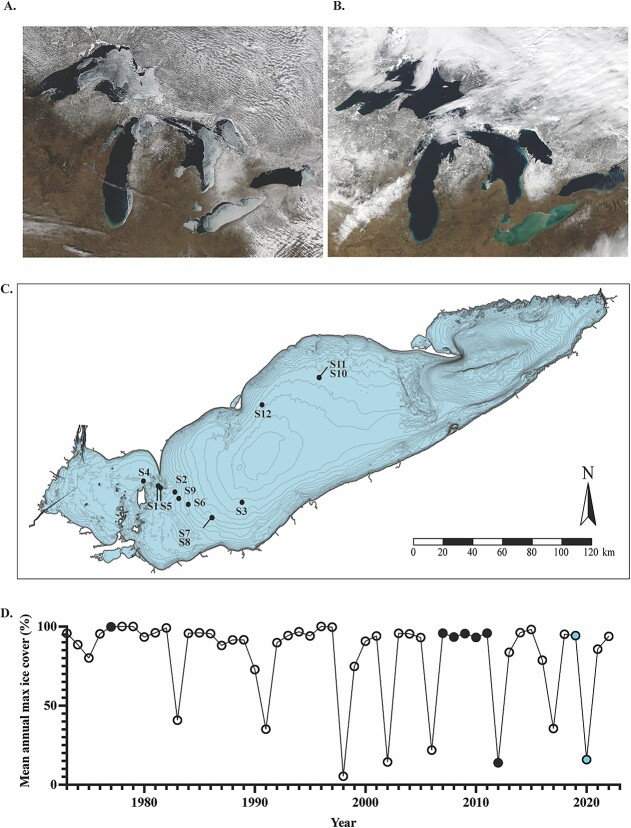
Climatic, spatial and temporal variability across Lake Erie samples. (A) MODIS satellite image (March 16th, 2014) depicting a large amount of ice cover across the Great Lakes. During the winter of 2014, Lake Erie had a mean annual ice cover of ~80%. (B) MODIS satellite image (February 12th, 2023) depicting a lack of ice cover across the Great Lakes. Sediment plumes can be observed throughout Lake Erie (the light brown coloration in the satellite image). During the winter of 2023, Lake Erie had a mean annual ice cover of ~8%. Figures adapted from data retrieved from the NOAA Great Lakes CoastWatch Node (NOAA 2023). (C) Sample sites across Lake Erie visited throughout winter-spring 2019 and 2020. (D) Historical trends in Lake Erie mean annual maximum ice cover (%). Open circles are years that (to our knowledge) do not have peer-reviewed published planktonic survey data. Solid black circles are years that were previously surveyed in prior published studies. Solid blue circles are years sampled in this study. Figure adapted from data retrieved from the NOAA GLERL database (NOAA-GLERL) [103].

Lake Erie and other lakes across the globe are experiencing declines in ice cover [6, 26, 27]. Projections suggest ice cover may disappear entirely across the Great Lakes by the end of the century [28]. This loss of ice cover presents a unique scenario for shallow lakes such as Erie (mean depth ~19 m). Due to predominant westerly winds blowing across the west-to-east axis of the lake, snow seldomly accumulates on the surface ice [4, 21, 29] (Figure 1A), allowing light to penetrate through ice cover to where diatoms are located. Indeed, a recent study found that light transmittance through wind-swept ice was 34%–43% in eutrophic lakes Minnetonka and Parker’s (Minnesota) compared to <1%–6% transmittance when the ice was snow covered [30]. This phenomenon has been further documented in other large lakes, such as Lake Michigan [29]. Yet, in the absence of ice cover, these wind patterns (and to a lesser extent convective mixing) create an isothermal water column with entrained sediment in shallow Lake Erie [31, 32] (Figure 1B). Indeed, Beall *et al*. [3] reported turbidities (in nephelometric turbidity units) a magnitude higher in ice-free Lake Erie in 2012 compared to the prior ice-covered year (2011) and noted that planktonic diatom abundances declined in the turbid water column. The findings of this study suggested that light limitation due to elevated turbidity within the ice-free lake was the key driver of diatom decline [3, 6, 33, 34].

We employed *in situ* analyses and metatranscriptomics to explore the hypothesis that winter diatoms are light limited in the ice-free water column. Facilitated by collaborative efforts with the US and Canadian Coast Guards [35], opportunistic samples were collected throughout 2019 and 2020, yielding samples collected from both ice-covered (2019) and ice-free (2020) water columns [36]. The survey also included spring samples, which we include for additional comparison. Our analyses suggest that ice cover alters planktonic diatom bloom magnitude and phylogeny while providing evidence of light limitation within diatom communities of the ice-free water column. We present evidence for two adaptations which we hypothesize increase the competitive fitness of some freshwater diatoms within the ice-free winter water column.

## Materials and methods

### Lake Erie winter–spring water column sampling

Samples of opportunity (*n* = 77) from the Lake Erie planktonic community were collected across temporal, spatial, and climatic gradients throughout the winter of 2019 and 2020 [36, 37]. This large-scale collaborative effort included multiple surveys conducted by the US Coast Guard Cutter *Neah Bay*, Canadian Coast Guard Ship *Limnos* and merchant vessel *Orange Apex*, resulting in a large metatranscriptomic dataset [36]. Prior to sample collection, water column physiochemical parameters were recorded along with meteorological conditions and ice cover observations. Briefly, water samples were collected from 0.5 m below the surface (thus referred to hereafter as “planktonic” because true “ice samples” were not collected). Samples were processed to provide estimates of dissolved and particulate nutrients (mg L^−1^, >0.2 μm), size-fractionated (>20 $\mathrm{\mu}$m and subsequently >0.22 $\mu$m) Chl *a* biomass ($\mathrm{\mu}$g L^−1^), phytoplankton taxonomy, and cell abundance (cells L^−1^). Samples were also collected for total community RNA. Measurement of Chl *a* was performed with fluorometry following extraction in 90% (vol/vol) acetone at −20°C [4]. Nutrients were measured by the National Center for Water Quality Research (Heidelberg University, Tiffin, OH, USA) using standardized techniques [38]. Samples of lake water were preserved with Lugol’s iodine and stored at room temperature until phytoplankton identification and enumeration. Briefly, samples were analyzed by counting phytoplankton in a measured aliquot by using a modified inverted microscope for the Utermöhl method plus a small magnification modification of the stratified counting technique of Munawar and Munawar [39]. A measured aliquot of mixed sample was placed into an inverted microscope counting chamber and allowed to settle for a minimum of 4 h per centimeter of overlying water depth. Larger and recognizable rare cells were counted at 400× along a minimum of one transect across the entire counting chamber. Smaller algae were counted at 1000× along a measured transect until a minimum of 300 cells were enumerated. Phytoplankton were counted as individual cells. All metadata are available online at the Biological and Chemical Oceanography Data Management Office [37]. Reference Supplemental Table 1 for further detail.

### RNA extraction and sequencing

RNA extractions were performed using previously described phenol-chloroform methods with ethanol precipitation [40]. Residual DNA in samples was digested *via* a modified version of the Turbo DNase protocol using the Turbo DNA-free kit (Ambion, Austin, TX, USA). Samples were determined to be DNA-free *via* the absence of a band in the agarose gel after polymerase chain reaction amplification using 16S rRNA primers as previously reported [36]. Samples were quantified by use of the Qubit RNA HS Assay Kit (Invitrogen, Waltham, MA, USA) and sent to the Department of Energy Joint Genome Institute for ribosomal RNA reduction and sequencing using a NovaSeq S4 2 × 151–nucleotide indexed run protocol (15 million 150-bp paired-end reads per library) as reported previously [36].

### Metatranscriptomic analysis

Bioinformatic filtering and trimming of raw reads was performed by the Department of Energy Joint Genome Institute using BBDuk (v.38.92) and BBMap (v.38.86) [41, 42]. Bioinformatic processing was conducted using an established metatranscriptomic workflow [43]. Trimmed and filtered libraries (*n* = 77) were concatenated and assembled (co-assembled) using MEGAHIT (v.1.2.9) [44]. Co-assembly statistics were determined *via* QUAST QC (v.5.0.2) [45]. Trimmed reads were mapped to the co-assembly using BBMap (default settings) (v.38.90) [41]. Gene predictions within the co-assembly were called using MetaGeneMark (v.3.38) [46] using the metagenome style model. Taxonomic annotations of predicted genes were determined using the MetaGeneMark protein file, EUKulele (v.1.0.6) [47] and the PhyloDB (v.1.076) database. This study highlights a challenge within the freshwater field at large: there is a lack of sequenced freshwater diatom taxa and an absence of freshwater annotated databases, which constrains evaluation of sequencing data. Indeed, Edgar *et al*. [9] noted only 23% of the taxonomic diatom annotations within their Lake Erie metatranscriptome could be tied to genera known to be present within the Great Lakes. Reavie [48] reiterated this gap, pointing out that there are numerous undescribed and unclassified diatom taxa. As a result, there may be transcriptional changes within the winter diatom community which have gone undetected within our study. Many of the annotations we generated best aligned to marine counterparts due to a lack of sequenced freshwater representatives (i.e. where reads are annotated as belonging to a genus “*-like*” genome). More broadly, sequence data are often not coincident with classic morphological taxonomy. Nonetheless, *A. islandica* filaments exhibit a distinct morphology from *Stephanodiscus* spp., and bioinformatic pipelines such as EUKulele accurately distinguish the taxonomy of these underrepresented eukaryotes at the class level [47]. Thus, this ambiguity in diatom taxonomy does not negate the observations in this study.

Genes were functionally annotated using eggNOG-mapper using a specified e-value of 1e^−10^ (v.2.1.7) [49], followed by the use of featureCounts [50] within the subread (v.2.0.1) package to tabulate read counts to predicted genes. Mapped reads were normalized to transcripts per million (TPM), representing relative “expression” values prior to statistical analyses (using ANOSIM, SIMPER, nMDS, *etc*.). To investigate transcriptional patterns of the winter diatom bloom community, we focused on a subset of libraries (*n* = 20) selected for consistency in sample collection methods (whole-water filtration) and diatom abundances (Supplemental Table 1). Thus, all data reported hereafter pertain to these 20 libraries. Raw data for all 77 transcriptomic libraries are available at the Joint Genome Institute Data Portal (https://data.jgi.doe.gov) under Proposal ID 503851 [36]. Please refer to the Supplemental Methods and Supplemental Table 1 for further details on approaches and all raw data information.

### Phylogenetic analyses

A phylogenetic tree of fasciclin containing domains (proteins of interest) was produced using differentially expressed (DE) putative proteins identified from this study (*n* = 18), domains recovered from the eggNOG ortholog database, and publicly available domains from NCBI [51]. A custom database was produced by downloading all NCBI diatom proteins using NCBI’s e-utilities language (*Bacillariophyta*; NCBI:txid2836). This was done to increase recruitment of diatom-specific sequences and reduce computing time needed to search against all NCBI entries. A DIAMOND (v.2.0.15) [52] blastp alignment was performed with putative fasciclin proteins and eggNOG domains against the diatom database to recover all putative diatom fasciclin domains. The recovered domains were then aligned (DIAMOND blastp) against the NCBI nonredundant database (nr). These results were compiled and collapsed to 80% similarity using CD-HIT (v.4.7) [53], and a multiple sequence alignment was performed using MAFFT (v.7.310) [54] with 500 iterations. Gaps were closed using trimAl with gappyout (v.1.4.rev15) [55] and examined using AliView (v.1.28) [56]. A phylogenetic tree (1000 bootstrap replicates) was constructed using IQ-TREE (v.2.2.0.3) with the built-in model test that selected for a general nonreversible Q matrix model estimated from the Pfam database (v.31) [57] with a gamma rate heterogeneity. The consensus tree was visualized using iTOL [58].

A phylogenetic tree of diatom proton-pumping rhodopsin containing domains (proteins of interest) was produced using DE (*n* = 2) and non-DE (*n* = 9) putative proteins identified from this study (total *n* = 11). NCBI nr putative proteins were searched using baited study sequences identified as rhodopsin/rhodopsin-like and with a diatom taxonomic designation by EUKulele *via* diamond BLASTp (v. 2.0.15). NCBI nr was also queried in the same way with potential freshwater diatom whole genomes with no suitable results. Retrieved amino acid sequences were collapsed at 100% using CD-HIT and aligned by MAFFT (v.7.520) with 1000 iterations. The alignment was then trimmed using trimAL (v.1.4. rev15) with the automated1 parameter. IQ-TREE (v. 2.2.0.3) was used to produce a consensus tree with 1000 bootstrap iterations using the built-in model test results (Q.pfam+G4 model). The resulting tree was modified from iTOL (v.6) visualization. Refer to Supplemental Methods and Supplemental Tables 1 and 2 for further details.

### Statistical analyses

Comparisons of water column physiochemical features by ice cover were made in Prism (v. 9.3.1) *via* two-tailed unpaired *t*-tests. Variability in expression (TPM) between transcriptomic libraries was assessed *via* ANalysis Of Similarities (ANOSIM) and Similarity Percentage (SIMPER) analyses using PRIMER (v.7) [59]. Bray–Curtis dissimilarity and nonmetric multidimensional scaling (nMDS) were performed in R (v.4.2.2). Differential expression of transcript abundance was performed using DESeq2 (v.1.28.1) [60]. Genes with an absolute log_2_ fold change (Log_2_FC) > 2 and adjusted *P* value of <.05 were considered differentially expressed; z-scores reported in heat maps were calculated by heatmapper.ca (clustering method: average linkage, distance measurement method: Pearson) [61]) using the DESeq2 variance stabilizing transformed values (VST) [61].

## Results

### Physiochemical profiles and winter community characterization

**Figure 2:**
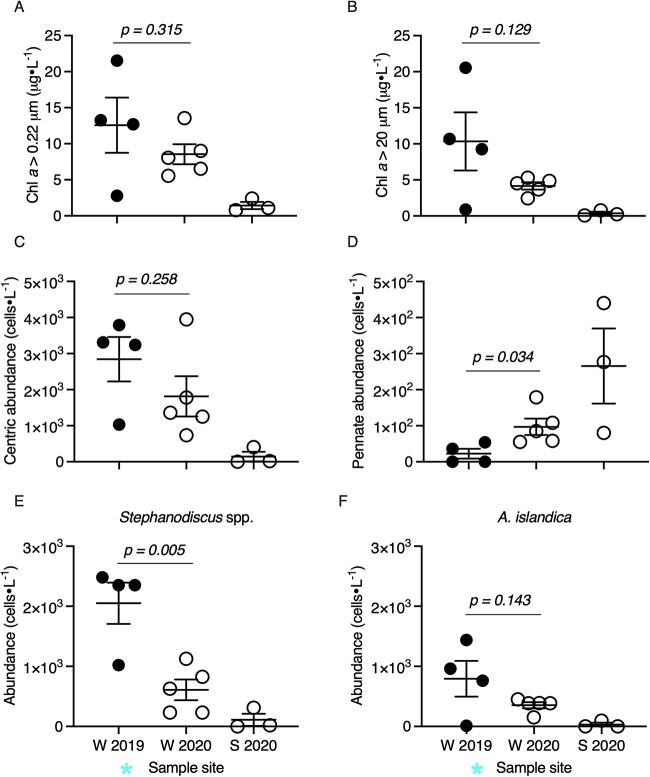
Characterization of biotic community across Lake Erie sample sites. Samples are organized on the x-axis by season (W, winter; S, spring) and year. Solid shapes indicate the sample was collected during ice cover (2019) open shapes indicate the sample was collected during no ice cover (2020). Ice cover samples are indicated by a blue asterisk. (A) Total Chl *a* concentration of the whole water column community (i.e. >0.22 $\mathrm{\mu} \text{m}\ \text{in}\ \text{size}$) ($\mathrm{\mu}$g L^−1^) (B) Chl *a* concentration of the large size fractioned community *(*i.e. >20 $\mathrm{\mu} \text{m}\ \text{in}\ \text{size}$) ($\mathrm{\mu}$g L^−1^). (C) Cell abundances (cells⋅L^−1^) of centric diatoms (*Stephanodiscus* spp. + *A. islandica* + small centric diatoms of 5–20 $\mathrm{\mu} \text{m}$). (D) Cell abundances of pennate diatoms (*Fragilaria* spp. + *Asterionella formosa* + *Nitzschia* spp). (E) Cell abundances (cells L^−1^) of *Stephanodiscus* spp., *Mediophyceae* class. (F) Cell abundances (cells ⋅L^−1^) of *A. islandica, Coscinodiscophyceae* class. Abbreviations: Chl *a*, chlorophyll *a.*

Planktonic water samples were collected at 12 sites throughout the central basin of Lake Erie with true biological replication at a subset of stations (Figure 1C) (Supplemental Table 1). Temporally, the samples span February–March 2019 and February–June 2020, yielding 14 winter and 6 spring libraries reported in this study. Climatically, the winter of 2019 was a year of high ice cover (mean maximum ice cover of ~81%), whereas winter 2020 was a year of negligible ice (mean maximum ice cover of ~20%) [62] (Figure 1D). Libraries 1–4 were collected during ice cover (ranging from 3 to 15 cm in thickness) while winter libraries 5–14 were collected during ice-free conditions (i.e. an absence of surface ice cover in that area). Winter lake surface temperatures ranged from ~0°C–6°C across sample sites (Supplemental Figure 1A). Nutrient concentrations at ice-covered sites were not significantly different from those at ice-free sites save for nitrate (Supplemental Figure 1B–H). While not significant (*P ≥* .129), the highest total Chl *a* concentrations (>0.22 $\mathrm{\mu}$m) of our samples (collected at 0.5 m depth) coincided with ice cover (Figure 2A and B). The larger sized-fraction of phytoplankton contributed ~70% (±27%) to total Chl *a* during ice cover and 50% (±13%) in ice-free winter sites (Supplemental Figure 2), but the differences were not significant (*P =* .202). Diatoms (*Bacillariophyta*) dominated the planktonic winter water column regardless of ice conditions, with other eukaryotic phytoplankton (e.g. *Chlorophyta*, *Cryptophyta*, and *Dinophyta*) present at concentrations 1–2 orders of magnitude lower (Supplemental Figure 3). While *Bacillariophyta* concentrations decreased slightly at ice-free sites (*P =* .326), *Dinophyta* concentrations significantly increased (*P =* .029), with *Cryptophyta* and *Chlorophyta* exhibiting similar trends (*P ≥* .054). Overall, centric diatoms (*Mediophyceae*, *Coscinodiscophyceae*) dominated the planktonic winter diatom community while pennate diatoms (*Bacillariophyceae*, *Fragilariophyceae*) were found at concentrations an order of magnitude lower (Figure 2C and D). Despite this dominance, centric diatoms demonstrated a decreasing trend in ice-free samples while pennate diatoms exhibited significant increases in ice-free samples (*P = .034*), albeit remaining at low abundances. Cell concentrations of the centric bloom formers *Stephanodiscus* spp. and *A. islandica* were highest during ice cover (Figure 2E and F). *Stephanodiscus* spp. concentrations were significantly higher than those of *A. islandica* in ice-covered samples (*P = .033*), yet not significantly greater than those of *A. islandica* in ice-free samples (*P = .194*) (Supplemental Figure 4). Further, while small centric diatoms (5–20 μm size) were not detected in ice-covered samples, they were found to range from ~300–3000 cells L^−1^ in ice-free samples (Supplemental Figure 5). These small centric diatom taxa accounted for ~83% of the winter diatom community at site 8, although they otherwise contributed an average of 26% to the total diatom community in ice-free samples (Supplemental Figure 6).

### Transcriptomic response of winter diatom community to ice cover

**Figure 3:**
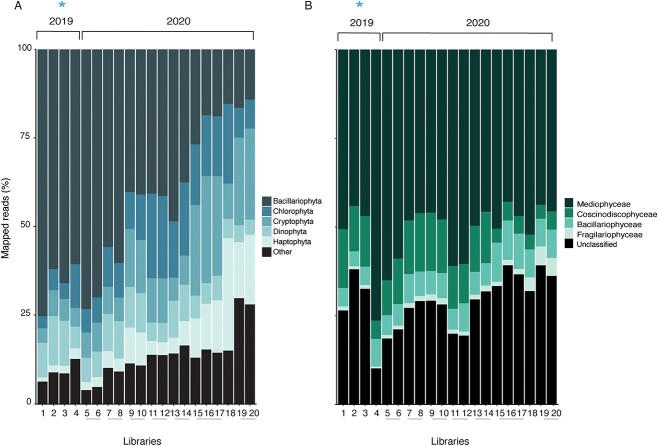
Relative transcript abundance of major eukaryotic phytoplankton taxa and diatom classes. Libraries are listed in chronological order of sample date on x-axes, with biological replicates joined by grey horizontal bars. Ice cover samples are indicated by a blue asterisk. (A) Relative transcript abundance of MEPT. All groups which formed <5% of the total mapped reads are included within “other” (*Amoebozoa*, *Hilomonadea*, *Excavata*, *Rhizaria*, not annotated [NA]). (B) Relative transcript abundance of *Bacillariophyta* classes *Mediophyceae*, *Coscinodiscophyceae*, *Bacillariophyceae*, *Fragilariophyceae*, and unclassified diatoms.

Diatoms dominated the transcript pool of major eukaryotic phytoplankton regardless of ice cover (Figure 3A). In turn, diatoms of the class *Mediophyceae* dominated diatom community transcription regardless of ice cover (Figure 3B). At the genus level of each class, there was a lack of definitive trends across libraries; thus, they are omitted from the main text (Supplemental Figures 7–10). Overall, there was no correlation between diatom cell abundance and transcript abundance (Supplemental Figure 11).

**Figure 4:**
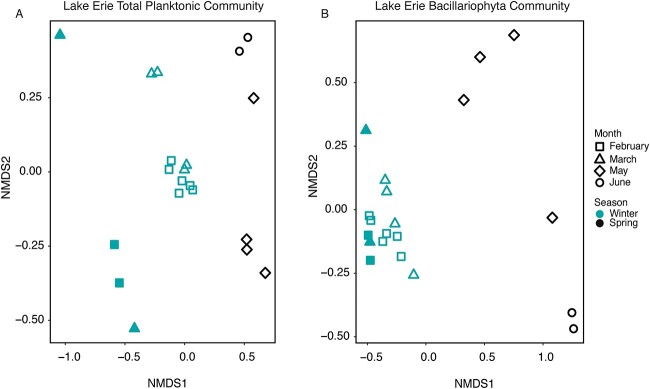
Dissimilarity (Bray-Curtis) clustering of the 20 metatranscriptomic library normalized expression values (TPM). (A) nMDS of the entire water column community expression, stress value = 0.063. (B) nMDS of the *Bacillariophyta* community expression, stress value = 0.050. Samples are presented as follows: February, squares; March, triangles; May, diamonds; June, circles. Blue indicates the sample was collected during the winter, black indicates the sample was collected during the spring. Solid shapes indicate the sample was collected during ice cover (2019) open shapes indicate the sample was collected during no ice cover (2020).

Normalized expression (TPM) profiles of the total water column community clustered by ice cover (Figure 4A), with SIMPER analyses demonstrating 64% dissimilarity between ice-covered and ice-free winter libraries (Supplemental Table 1). ANOSIM tests confirmed that ice strongly affected winter community gene expression (*r = 0.87, P = .002*) (Supplemental Figure 12A) (Supplemental Table 1). Surprisingly, diatom community expression did not cluster as strongly by ice cover (Figure 4B), with SIMPER analyses indicating an average dissimilarity of 47% between ice-covered and ice-free libraries (Supplemental Table 1). ANOSIM tests confirmed that ice cover exerts a lesser influence on winter diatom community expression overall than on the full water column community (*r =* 0.28*, P =* .059) (Supplemental Figure 12B). In contrast, season had a strong effect on diatom expression (SIMPER average dissimilarity = 77%; ANOSIM *r =* 0.93*, P =* .001) (Supplemental Table 1).

To investigate how ice cover contributed to the dissimilarity in winter diatom expression, DE analyses were performed. In total, 354 genes belonging to putative *Bacillariophyta* were differentially expressed *(|Log_2_FC| ≥* 2*, P_adj_ <* .050), with 311 of these genes increasing in relative expression in ice-free samples (variable of interest) and 43 decreasing (Supplemental Table 1). Diatoms of the class *Mediophyceae* had the highest representation, comprising ~50% of DE genes, while other classes formed a net total of ~10% (40% unclassified diatoms) (Supplemental Figure 13A). Further analysis revealed 33% of the polar centric DE genes were annotated as *Chaetoceros*-like (Supplemental Figure 13B), despite *Chaetoceros*-like genes forming $\le$10% of mapped reads throughout the winter libraries (Supplemental Figure 7). Here, the “*Chaetoceros*-like” label arises because the transcriptomes were annotated with largely marine-comprised databases due to a lack of comprehensive freshwater taxonomic sequencing [9, 63]. Hence, diatom classes are reported in the text and genera are reported in the Supplemental Figures and Supplemental Table 1.

**Figure 5:**
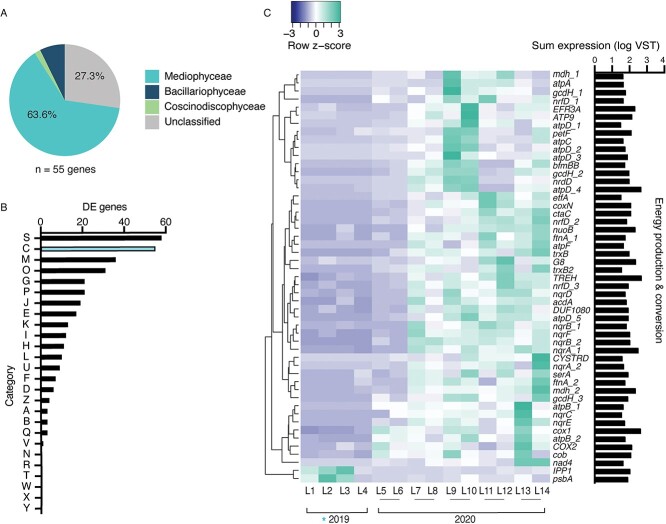
*Bacillariophyta* energy production and conversion transcript abundance patterns in response to ice cover. A) Taxonomic distribution of DE genes categorized within COG category C (energy production and conversion). (B) COG assignments for all 354 DE genes in response to ice cover, with COG category C indicated in blue. (C) Heatmap depicting COG category C DE gene expression (VST) in response to ice cover across the 14 winter libraries.

**Figure 6:**
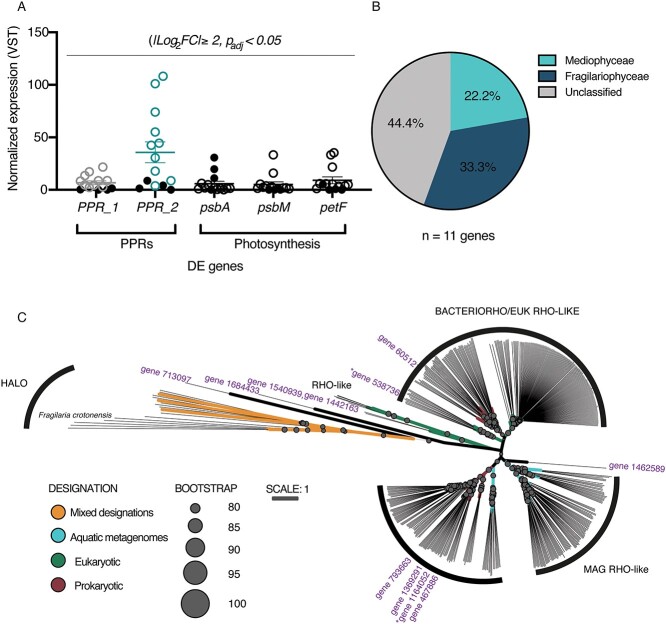
*Bacillariophyta* PPR transcript abundance patterns in response to ice. (A) Normalized expression (VST) of two DE genes functionally annotated as PPRs (PPR_1 [grey], PPR_2 [teal]) and representive DE genes functionally annotated to be involved in photosynthesis (black). Photosynthetic genes were selected because they encode for photosynthetic reaction centers (*psb*A, *psb*M) or the transfer of electrons along the photosystems (*pet*F). Each circle corresponds to gene expression in one of the 14 libraries. Solid black circles indicate the sample was collected during ice cover (2019) open shapes indicate the sample was collected during no ice cover (2020). (B) Taxonomic distribution of the 11 genes functionally annotated as PPRs (and confirmed with subsequent phylogenetic analysis). (C) Phylogenetic tree of PPR distribution within diatoms. Putative rhodopsin-like proteins (*n* = 11, purple) were distributed within several rhodopsin groups and sub-groups to determine likelihood of putative genes being of bacterial or eukaryotic origins. The position of study genes is labelled by their associated groups, with the exception of genes 713 097 and 1 684 433 being GCPR transmembrane rhodopsin associated proteins, and gene 1 462 589 being unclear (most closely associated with the genes of metagenomic origin. Bootstrap values are based off 1000 replicates and are identified if above 80. Abbreviations: BACTERIORHO/EUK RHO-LIKE; bacteriorhodopsins and eukaryotic origin rhodopsin-like putative proteins, HALO; halorhodopsin, METAGENOME RHO-LIKE; metagenomic origin rhodopsin-like putative proteins, RHO-like; sensory eukaryotic rhodopsin-like proteins, XANTHO; xanthorhodopsin. The two DE PPRs are indicated with asterisks.

Genes categorized in COG category C (Energy production and conversion) were the second most highly represented category within the DE dataset, with most genes exhibiting increased expression within planktonic ice-free diatom communities (Figure 5). Of these genes, 64% belonged to the class *Mediophyceae* (Figure 5A, Supplemental Figure 14). The expression of genes encoding for iron-containing photosynthetic proteins (ferrodoxin-*petF*, flavoprotein-*etfA*, and ferritin-*ftnA*) increased in relative expression in ice-free communities, while expression of photosystem II-*psbA* decreased (Figure 5B and C). Likewise, relative expression of genes within COG category P (inorganic ion transport and metabolism) increased in ice-free samples (Supplemental Figure 15A), with expression of putative iron transporting genes (OMFeT_1–3) increasing in ice-free communities. The DE genes within COG category P also largely belong to the *Mediophyceae* class, comprising ~40% of the annotated genes (Supplemental Figures 15 and 16). Two proton-pumping rhodopsin genes (PPRs; COG category S), which were most recently found to be a light-driven, retinal-based alternative to classical phototrophy in a cold-adapted freshwater photosynthetic bacterium [64], significantly increased in expression within planktonic ice-free diatom communities (Figure 6A, Supplemental Figure 17A). Further, the expression of 9 additional diatom PPRs increased within the ice-free water column, though they fell short of our statistical differential expression cutoff (Supplemental Figure 17B). Taxonomic annotations demonstrated these 11 PPRs largely belonged to the *Fragilariophyceae* (~33%) and *Mediophyceae* (~22%) classes (unclassified = ~44%), with the two DE PPRs annotated at the phylum (PPR_1, *Bacillariophyta*) and genus level (PPR_2, *Pseudo-nitzschia fraudulenta*–like) (Figure 6B). Phylogenetic analyses suggested diatoms horizontally acquired PPRs from bacteria, as there is evidence for at least three instances of horizontal gene transfer within our analysis (Figure 6C). The majority of the PPRs in our study clustered with or near eukaryotic rhodopsins. The most highly DE PPR in our study (PPR_2, gene 538 736) clustered closely with the marine diatom PPR belonging to *Pseudo-niztschia granii* (Figure 6C) [65, 66].

**Figure 7:**
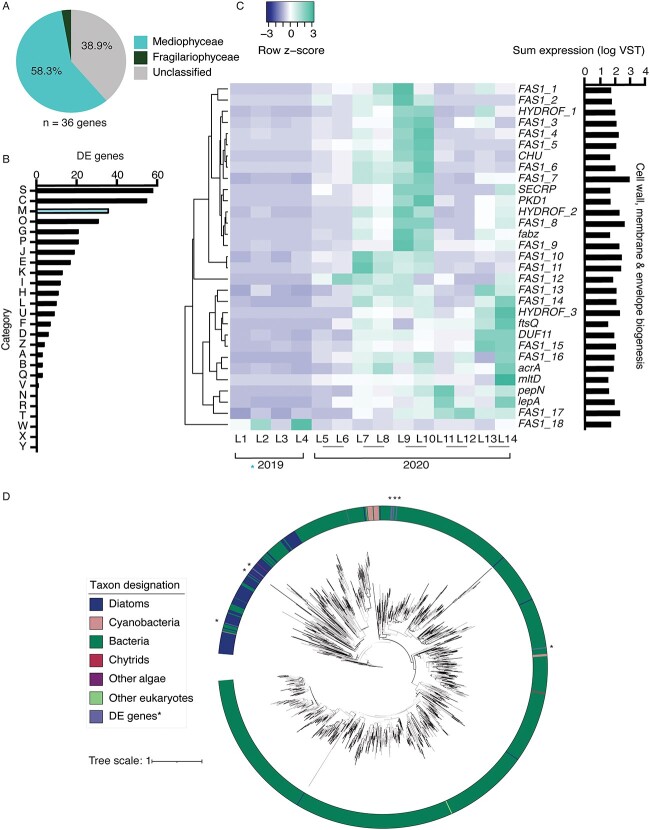
*Bacillariophyta* fasciclin transcript abundance patterns in response to ice cover. (A) Taxonomic distribution of DE genes categorized within COG category M (cell wall, membrane, envelope biogenesis). (B) COG assignments for all 354 DE genes in response to ice cover, with COG category M indicated in blue. (C) Heatmap depicting COG category M DE gene expression (VST) in response to ice cover across the 14 winter libraries. (D) Phylogenetic tree of fasciclin distribution within diatoms. Bootstrap values above 70 are indicated with black lines. The FAS1 domain was found in 141 marine and freshwater diatoms of diverse ecological habitats (indicated in blue). The 18 DE diatom fasciclins in this study are indicated in purple with asterisks. Bacterial fasciclins are indicated in dark green, with cyanobacteria (pink), chytrids (red), other algae (purple), and other eukaryotes (light green).

DE analyses in response to season were performed with diatom libraries to identify trends truly unique to the ice cover DE dataset. The top 10 COG categories represented in each dataset overlapped, except for COG category M, which was the 3rd most abundant in ice cover analyses compared to the 12th most abundant in season analyses (Supplemental Figure 18). Further analysis of these COG M (Cell wall, membrane, and envelope biogenesis) genes revealed 58% belonged to the class *Mediophyceae* (Figure 7A, Supplemental Figure 19). Fifty percent of the DE COG M genes encode for fasciclins (FAS1), which increased in expression under ice-free conditions (Figure 7B and C). Fasciclins are secreted glycoproteins involved in diatom cell–cell adhesion and cell–extracellular matrix adhesion [67, 68]. All 18 DE fasciclin genes were either assigned to the class *Mediophyceae* or unclassified beyond the phylum level (*Bacillariophyta*). Phylogenetic analyses indicated that diatoms horizontally acquired FAS1 from bacteria, as there is evidence for at least six instances of horizontal gene transfer within our analysis (Figure 7D). Broadly, the FAS1 domain is widely distributed in diatoms, with ~140 marine and freshwater diatoms found to contain this protein domain, including the model cold-adapted diatom *Fragilariopsis cylindrus* [69].

## Discussion

In the present study we examined how planktonic communities (specifically diatoms) responded to declining ice cover in a northern temperate lake (Lake Erie). Ice cover has declined by ~70% on the Laurentian Great Lakes over the 45-year period of 1973–2017 [70]. It has been suggested this decline in ice cover increases light limitation in shallow lakes such as Lake Erie; hence, we investigated this hypothesis that diatoms are light limited in the turbid ice-free water column [3]. In this study, we found evidence suggesting that ice-free conditions decreased planktonic diatom bloom magnitude and altered composition. Diatoms exhibited increased relative expression of photosynthesis and iron-transport genes under ice-free conditions: trends which are consistent with light limitation [71, 72]. Further, metatranscriptomic analysis provided evidence for two novel hypotheses concerning diatom adaptations to the ice-free state of the lake: (i) the ice cover PPR energy hypothesis and (ii) the fasciclin mediated rafting hypothesis. We provide this information couched within the context of the ecophysiological implications of a climatically altered future for psychrophilic aquatic communities.

### Ice-free conditions alter planktonic diatom bloom magnitude and composition

It was previously noted that planktonic biovolumes of *A. islandica* were 95% decreased in the ice-free turbid water column (2012) compared to the ice-covered conditions during the years prior (2010, 2011), with light limitation cited as the potential driver of this trend [3]. By comparison, planktonic Chl *a* biomass, centric diatom counts, and *A. islandica* counts did not significantly decrease relative to ice cover in our study, though they all exhibited a consistent declining trend. However, our findings are in juxtaposition to those of a prior Lake Erie winter study (2007–2010), which reported planktonic abundances of *A. islandica* and *Stephanodiscus* spp. 1–2 magnitudes higher, with Chl *a* concentrations supporting this trend [4]. In addition, subsequent studies also reported planktonic diatom communities overwhelmingly dominated by *A. islandica,* with *Stephanodiscus spp.* present in lesser concentrations [4, 9–11, 73]. In contrast, we discovered that planktonic cell abundances of *Stephanodiscus* spp. were significantly higher than those of *A. islandica* in the ice-covered community. We hypothesize that a decrease in consecutive years of high ice cover may drive this decline of *A. islandica* dominance (Figure 1D). In addition, we suggest that the presence/absence of ice cover alters the location of diatoms throughout the water column, though comprehensive vertical profiles of the water column will be required to address this theory moving forward. Regardless, our results suggest that the winter planktonic diatom bloom community has markedly declined in magnitude and altered in composition since prior winter Lake Erie surveys (2007–2012).

In turn, it has been suggested that declines in diatom biomass may cause this niche to be filled by cryptophytes and dinoflagellates, as mixotrophs are suggested to be better suited for the turbid water column [3, 6]. While we observed significantly higher abundances of these groups in ice-free samples within our study, their cellular and transcriptional abundances remained below those of centric diatoms by an order of magnitude. Hence, our results demonstrate that low ice cover during this season did not induce significant large-scale phyla-level shifts in major eukaryotic phytoplankton community composition (within planktonic samples) as previously suggested. Cumulatively, these findings imply that future ice-free winter communities may remain dominated by centric diatoms as observed in this study, albeit at a lesser magnitude.

While net centric diatom abundances did not significantly differ by ice conditions in our study, diatom community composition exhibited significant changes at the genus level. Cell abundances of *Stephanodiscus* spp. were ~50% lower in ice-free samples, resulting in approximately equal abundances of *Stephanodiscus* spp. and *A. islandica* within the ice-free water column. Further, small centric diatom taxa (5–20 μm size) were mainly absent in water column samples from ice-covered sites yet formed 10%–82% of total diatom counts in ice-free sites, with a bloom of these taxa noted at site 8. These findings suggest that ice-free conditions may increase populations of smaller, centric diatoms in future warmer and ice-free winters. This trend is supported by prior studies which demonstrated that warming temperatures decrease phytoplankton cell size [74] and select for smaller taxa [75, 76]. We also noted significant increases in pennate diatom abundance in the ice-free water column. Cumulatively, if these observations represent long-term trends, future ice-free diatom communities will be more diverse with lower biomass.

### Evidence of light limitation within the ice-free water column

Prior studies have demonstrated that ice cover inherently alters underwater light regimes [30, 77]. Here, we provide preliminary evidence of light limitation within the turbid, ice-free Lake Erie water column. The relative expression of photosynthesis-associated genes increased overall within ice-free diatom communities, suggesting potential efforts to increase light capture and light-driven processes within the turbid water column. We observed an increase in expression of iron transporters coinciding with various genes encoding for iron-rich photosynthetic structures and photosystem components. In support of our findings, a prior study found temperate phytoplankton acclimate to low-light conditions by increasing their number of iron-rich photosystems [72]. Thus, our data suggest that freshwater diatoms in the ice-free, turbid Lake Erie water column may have been attempting to build additional photosystems in response to decreased light availability. Building upon this finding, our data offer transcriptional support for a prior study which found primary production rates (measured by ^14^C-bicarbonate incubations) to be lower within the Lake Erie ice-free water column (2012) compared to the ice-covered water column (2010–2011) [3]. Further, the expression of the two diatom PPRs within our dataset significantly increased within the ice-free water column, whereas those involved in classical photosynthesis exhibited a net decline, suggesting that diatoms may be attempting to use alternative phototrophic strategies in addition to classical photosynthesis within the ice-free water column. Other studies have demonstrated that enlarged light antennae are another response to light limitation, as this increases light harvesting [78] and is suggested to be particularly advantageous in cold environments [71]. While we did not observe evidence of this phenomenon in our dataset, it may have occurred prior to or after sampling of the community, as metatranscriptomics offers only a “snapshot” episodic glimpse at community response. In addition, we note that transcription does not always indicate translation. Hence, while we found transcriptomic supportive evidence of light limitation in this study, further research and physiological confirmation is required.

### Role of PPRs as a function of ice cover

We observed increases in the expression of genes encoding for PPRs within the ice-free diatom community. PPRs are light-harvesting, retinal-containing proton pumps distinct from the chlorophyll-containing antenna of classical photosynthesis [79], yet capable of absorbing as much light energy as Chl *a* [80]. On a global scale, it is thought that microbial rhodopsin-driven phototrophy is a major marine light harvesting process [81]. Beyond prokaryotes, PPRs have been characterized within a number of marine diatoms [65, 82] and dinoflagellates [83] and have been suggested to serve as an alternative light-driven energy source for marine diatoms under conditions which limit classical photosynthesis [65, 82, 84]. However, compared to studies in the marine literature [80, 83, 85–88], PPRs are understudied in fresh waters. Yet, it has recently been suggested that PPRs may play a role in fresh waters: a photoheterotrophic bacterium isolated from an alpine lake used PPRs as an alternative phototrophy mechanism [64]. The authors of that study hypothesized that the contribution of PPRs to energy generation was linked to ice cover. Here, we present evidence that diatoms within the ice-free Lake Erie water column increase the expression of PPRs in the ice-free, low-light turbid environment, lending support to hypotheses regarding an ecophysiological role of PPRs within icy freshwater environments.

There are a variety of potential ecophysiological explanations for the PPR phenomenon. For example, recent evidence indicates that ice cover exerts a profound effect on light intensity and spectral signatures in the winter water column [30]. The use of PPRs may be an attempt to optimize the harvest of wavelengths beyond those absorbed by Chl *a*; lending further evidence to the effects of reduced light. Indeed, PPRs absorb at a maximum wavelength of ~525 nm (green light) [86] in contrast to Chl *a*, which absorbs at 430-470 nm (blue) and 660-670 nm (red) wavelengths. While diatoms are adept at scavenging blue-green wavelengths using fucoxanthin and chlorophyll c within their photosynthetic light harvesting complexes [9, 89], polar marine studies suggest PPRs might be favored at cold temperatures when photosynthesis is slowed [82, 90–92]. Hence, PPRs may allow diatoms to access alterative light niches in the cold, turbid water column. This is further supported in our data, as expression of two diatom PPRs was significantly higher in the ice-free water column whereas the expression of *psb*A was significantly higher in ice-covered samples (Figure 6A). Alternatively, green and blue light are less attenuated within the water column than other wavelengths. Hence, PPRs may be involved in light-acquisition during well-mixed isothermal conditions when diatoms would be mixing throughout the benthic and pelagic environments in shallow lakes (Supplemental Figure 20). In contrast, in some systems PPRs may also be critical within the snow-free, ice-covered water column. A recent study determined that the dominant wavelength at 0 m under cleared ice ranged from 486 to 589 nm (blue-green light) within two Minnesota lakes [30]. Cumulatively, these findings imply that there are ecophysiological role(s) of PPRs for diatoms across global freshwater and marine environments. Thus, our observations suggest that the role of these proton-pumping rhodopsins within fresh waters demands more attention [93], as it is possible that in future scenarios (less ice cover, more turbidity) they may serve as important evolutionary selectors.

### Role of fasciclins in the ice-free turbid water column

**Figure 8:**
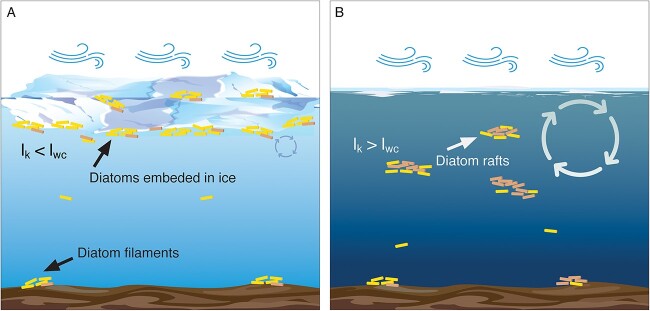
Schematic representation of how ice cover alters freshwater diatom colocation strategy throughout the water column. (A) Ice-covered water column exhibiting minimal convective mixing. As a result, I_k_ < I_wc_ (where I_k_ = irradiance at which photosynthesis is light saturated and I_wc_ = mean water column irradiance). I_wc_ is calculated based on light extinction coefficient and mixing depth, which in an ice-free winter (i.e. holomictic state) is the bottom, while in the presence of ice-cover, is limited to shallow convective mixing. Diatoms partition to the surface ice cover within the photic zone. (B) Ice-free water column which exhibits isothermal conditions and thorough mixing. As a result, I_k_ > I_wc_, and light-limited diatoms express fasciclins to form rafts of increased buoyancy to optimize their ability to harvest light in the turbid water column. Beige colored diatoms in both diagrams represent diatoms that possess PPRs, with an increased number of PPR-possessing diatoms selected for in the ice-free turbid water column in panel B.

Though fasciclins (FAS1) remain widely uncharacterized in diatoms, prior studies have described fasciclins within the diatom species *Amphora coffeaeformis* [67] and *Phaeodactylum tricornutum* [68]. Both studies identified fasciclin proteins within diatom-secreted exopolymer substance adhesion trails and concluded these molecules facilitate diatom motility, adhesion and aggregation. In this study, 58% of the DE diatom fasciclins belonged to the class *Mediophyceae* (27% to unclassified diatoms). As a result, we hypothesize *Mediophyceae* diatoms were rafting *via* cell-adhesion fasciclins to optimize their location within the ice-free, turbid Lake Erie water column, thus avoiding light limitation (Figure 8).

Our hypothesis is largely based on a similar “rafting” strategy that is well-documented in centric marine diatoms such as *Rhizosolenia* spp. [94–97].These studies demonstrated that rafts become positively buoyant in response to a variety of physiological stressors [96, 98]. Hence, there are likely additional benefits this rafting behavior incurs beyond light acquisition (i.e. defense against grazers, evasion of nutrient limitations, etc.), which merit further attention. More broadly, our phylogenetic analyses suggest this fasciclin-mediated rafting hypothesis may not be unique to Lake Erie diatoms alone. Fasciclins were identified in ~140 marine and freshwater diatoms, including the model polar marine diatom *F. cylindrus* [69]. Hence, this further implies an ecophysiological role exists for these proteins in the globally frigid waters, which in turn indicates that further research is required regarding the role of fasciclins within polar aquatic systems and psychrophilic organisms broadly, especially when considering the rapid global decline in ice cover.

## Conclusions

Lakes are sentinels of climate change [99]. Indeed, our study builds on data which demonstrated that community-wide responses to declines in ice cover are already in place. We provide evidence which suggests diatom changes may be driven by light limitation in the turbid ice-free water column. Indeed, Ozersky *et al*. [6] suggested warmer winters will induce a change in the Great Lakes mixing regime, shifting from dimictic mixing patterns to a warm monomictic mixing pattern characterized by continuous isothermal conditions throughout winter. Hence, adaptations to evade coinciding exacerbations in light limitation (such as the possession of PPRs and FAS1 described in this study) may be of increased importance in future winter diatom survival as phytoplankton adapt to ice-free winters. Indeed, our data suggests climate change may not be just a “temperature” problem in the case of shallow temperate lakes, but a “light” problem. Regardless, with diatoms previously described as “one of the most rapidly evolving eukaryotic taxa on Earth” [100, 101] and prone to promiscuous horizontal gene transfer events [102], it would be surprising if they failed to adapt to an ice-free future. Ultimately, we cannot place the metatranscriptomic observations we describe in a quantitative framework. To this end, our observations, which demonstrate variability associated with conditions consistent with projected future climate scenarios, carve out a critical path forward and provide cautionary insight of what may be yet to come in global temperate lakes.

## Supplementary Material

Supplementary_Tables_1-2_wrad015

Zepernick_et_al_2023-Supplemental_Figures_wrad015

Zepernick_et_al_2023_Supplemental_Methods_NEW_copy_(1)_wrad015

## Data Availability

Raw and processed reads for the data used in this study are available through the JGI Data Portal (https://data.jgi.doe.gov) under Proposal ID 503851.
